# The Dual NOD1/NOD2 Agonism of Muropeptides Containing a Meso-Diaminopimelic Acid Residue

**DOI:** 10.1371/journal.pone.0160784

**Published:** 2016-08-11

**Authors:** Yulia A. Dagil, Nikolai P. Arbatsky, Biana I. Alkhazova, Vyacheslav L. L’vov, Dmitriy V. Mazurov, Mikhail V. Pashenkov

**Affiliations:** 1 Laboratory of Clinical Immunology, National Research Center “Institute of Immunology of the Federal Medical-Biological Agency”, Moscow, Russia; 2 Laboratory of Preparative Biochemistry, National Research Center “Institute of Immunology of the Federal Medical-Biological Agency”, Moscow, Russia; 3 Laboratory of Immunochemistry, National Research Center “Institute of Immunology of the Federal Medical-Biological Agency”, Moscow, Russia; Universitat Hohenheim, GERMANY

## Abstract

Muropeptides are fragments of peptidoglycan that trigger innate immune responses by activating nucleotide-binding oligomerization domain (NOD) 1 and NOD2. Muropeptides from Gram-negative bacteria contain a meso-diaminopimelic acid (meso-DAP) residue in either a terminal or a non-terminal position. While the former ones are known to be recognized by NOD1, much less is known about recognition of muropeptides with non-terminal meso-DAP, which are most abundant moieties of Gram-negative peptidoglycans. Here, we developed a novel system to assess biological activity of muropeptides, based on CRISPR/Cas9-mediated knockout (KO) of *NOD1* and *NOD2* genes in modified HEK293T cells. Using NOD1/NOD2 knockout and overexpression systems, as well as human monocytes and macrophages, we refine the current view of muropeptide recognition. We show that NOD2 can recognize different natural muropeptides containing a meso-DAP residue (preferably in a non-terminal position), provided they are present at micromolar concentrations. NOD2 accepts muropeptides with long and branched peptide chains and requires an intact N-acetylmuramyl residue. Muropeptides with non-terminal meso-DAP can activate NOD1 as well, but, in this case, probably require peptidase pre-processing to expose the meso-DAP residue. Depending on NOD1/NOD2 ratio in specific cell types, meso-DAP-containing muropeptides can be recognized either primarily via NOD2 (in monocytes) or via NOD1 (in monocyte-derived macrophages and HEK293T-derived cells). The dual NOD1/NOD2 agonism of meso-DAP-containing muropeptides should be taken into account when assessing cellular responses to muropeptides and designing muropeptide immunostimulants and vaccine adjuvants.

## Introduction

Peptidoglycan (PG) is a major microbe-associated molecular pattern of bacterial origin and a potent activator of innate immune responses [1, 2]. PG consists of long polysaccharide chains, composed of alternating residues of N-acetyl glucosamine (GlcNAc) and N-acetyl muramic acid (MurNAc), and short peptides, usually 3–5 amino acids long, which are covalently linked to MurNAc. In Gram-negative bacteria, third positions in these oligopeptides are occupied by meso-diaminopimelic acid (meso-DAP) [3]. The free NH_2_-groups of meso-DAP can form covalent linkages with C-termini of peptides stemming from neighbouring polysaccharide chains, creating inter-chain bridges important for the rigidity of the PG envelope [3].

**Fig 1:**
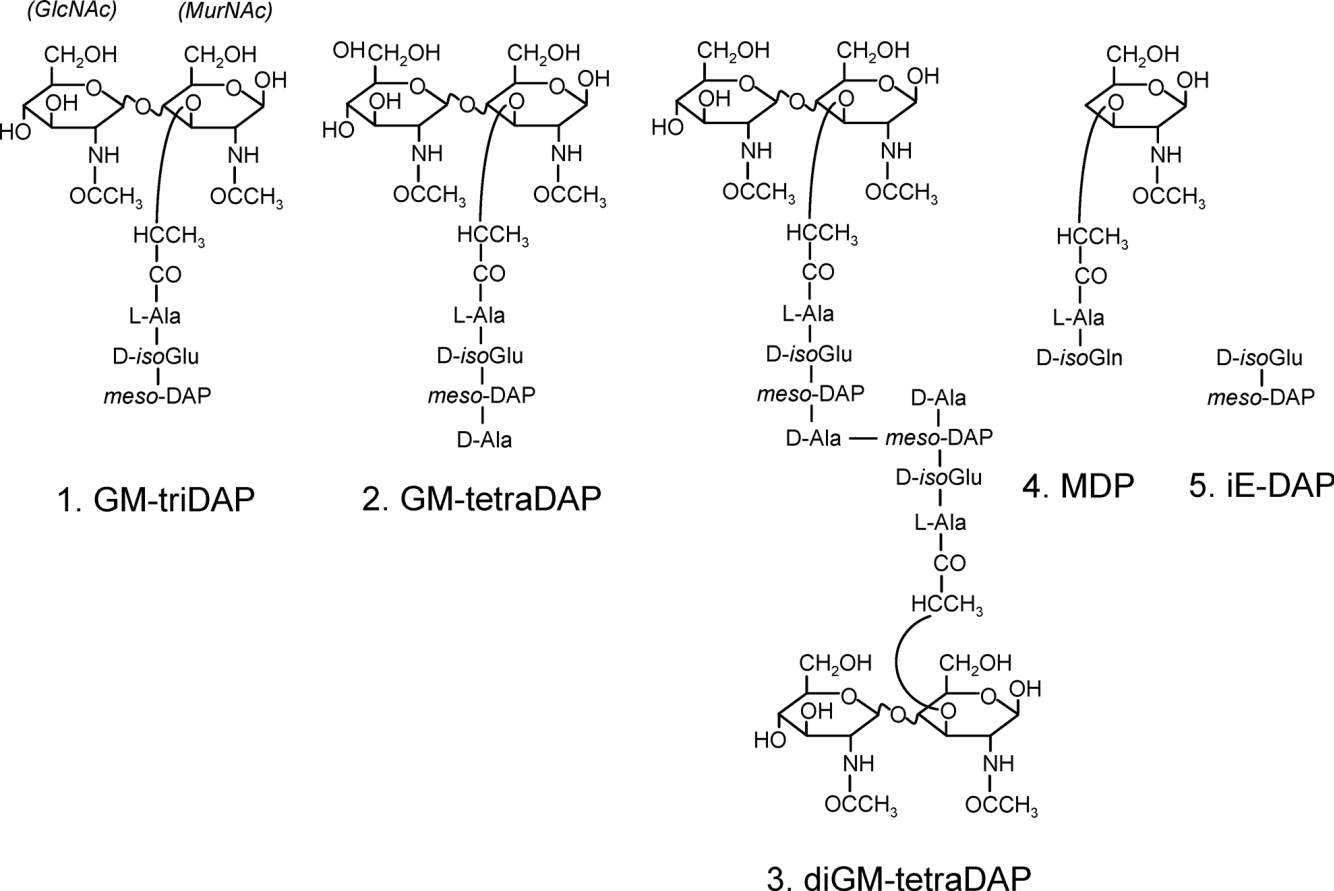
Structural formulas of muropeptides used in the study.

The innate immune system recognizes PG mainly in the form of muropeptides, which arise either due to cleavage of the MurNAc → GlcNAc glycoside bonds by bacterial or host enzymes such as lysozyme, or in the course of PG biosynthesis [4, 5]. Natural muropeptides consist of the GlcNAc–MurNAc (GM) disaccharide and the oligopeptide chains (Fig 1). Muropeptides trigger two cytosolic receptors, nucleotide-binding oligomerization domain (NOD) 1 and NOD2 [6, 7], resulting in the activation of the nuclear factor (NF)-κB and expression of NF-κB-regulated genes [7–9], as well as in a number of other biological responses [10–14]. Currently, NOD1 is thought to recognize muropeptides with C-terminal meso-DAP, such as GlcNAc–MurNAc–L-Ala–D-isoGlu–meso-DAP (GM-triDAP) or MurNAc–L-Ala–D-isoGlu–meso-DAP (M-triDAP) [6, 8, 15] (Fig 1). These muropeptides are specific for Gram-negative bacteria. NOD2 is viewed as a more general bacterial sensor, because it can recognize muramyl dipeptide (MDP) [7, 15], which can be derived from most types of PGs by complex enzyme treatment [16]. NOD2 also recognizes several Gram-positive muropeptides, but is thought not to recognize Gram-negative muropeptides containing meso-DAP [15, 17].

However, in the natural PGs, GM-triDAP represents a relatively rare building unit, and units equivalent of MDP are usually absent [3, 16]. In Gram-negative PGs, the most frequent unit is GM-tetraDAP (GlcNAc–MurNAc–L-Ala–D-isoGlu–meso-DAP–D-Ala) [3]. Furthermore, because of inter-chain bridges, dimeric muropeptides can be formed upon lysozyme digestion of PG. A frequently found dimer is diGM-tetraDAP, wherein the terminal D-Ala of one GM-tetraDAP monomer is linked, by an amide bond, to the ω-amino group of meso-DAP of the other GM-tetraDAP monomer (Fig 1). Data on innate immune recognition of these muropeptides, where meso-DAP is non-terminal, are controversial. For example, according to two independent groups, M-tetraDAP is a strong [17] and a weak [18] NOD1 agonist, while both groups concur that M-tetraDAP does not activate NOD2 [17, 18]. There are no data on NOD1/NOD2 agonism of diGM-tetraDAP, except for one brief statement on the lack of its activity towards NOD1 [8]. We showed that both GM-tetraDAP and diGM-tetraDAP can activate human macrophages, albeit less efficiently than GM-triDAP [19], but mechanisms of activation were not investigated. Furthermore, there has been a controversy regarding a subset of patients with Crohn’s disease who have an inactivating NOD2 mutation and do not respond to MDP: one group reported that PBMC from these patients do not respond to NOD1 agonists as well [20], contrary to a report from another group [21]. These discrepancies raise concerns as to the full validity of the current model of muropeptide recognition.

To assess the specificity of muropeptides towards NOD1 or NOD2, a model is required where only one of the two NOD receptors would be activated at a time, without interference from each other and from third-party receptors. A widely used in-vitro model is based on HEK293T cells, which are transfected with NOD1 or NOD2 expression vectors together with a vector encoding a reporter gene under an NF-κB-inducible promoter [6, 8, 15, 17, 18, 22]. Muropeptides are either delivered intracellularly together with the vectors or added afterwards. However, NOD1 and NOD2 expression vectors themselves induce NF-κB activation [23, 24], which necessitates their careful titration. Furthermore, HEK293T cells endogenously express at least NOD1 and can be activated by NOD1 agonists even if not transfected with NOD1 [25, 26], which may result in underestimation of responses to muropeptides. Cells from *Nod1-* or *Nod2*-knockout (KO) mice represent a neater model, but cannot substitute for human cells because of known inter-species differences in muropeptide recognition [18].

Here, we developed a novel in-vitro model to assess agonistic properties of muropeptides. Instead of overexpressing NOD1 or NOD2 in HEK293T cells, we permanently knocked out endogenous NOD1 and/or NOD2 in these cells using the recently developed CRISPR-Cas9 technology [27]. The technology is based on the simultaneous transgenic expression of a single-guide RNA (sgRNA) hybridizing with the target genomic sequence, and Cas9 nuclease that recognizes sgRNA and creates a double-stranded DNA break at that site, which results in indel mutations and loss of gene function [27]. Using both the new and the ‘standard’ experimental systems, we show that human NOD2 can recognize meso-DAP-containing muropeptides such as GM-triDAP, GM-tetraDAP and diGM-tetraDAP, provided they are given at sufficiently high concentrations and carry an intact MurNAc residue. GM-tetraDAP and diGM-tetraDAP can activate NOD1 as well, but probably require pre-processing into GM-triDAP or triDAP. We further show that NOD2 recognition of meso-DAP-containing muropeptides is especially important in monocytes, where expression of NOD2 prevails over that of NOD1.

## Materials and Methods

### Plasmids

The pGL4.32[luc2P/NF-kB-RE/Hygro] plasmid, which encodes a human-codon-optimized luciferase gene (luc2P) under an NF-κB-dependent promoter, was purchased from Promega (Madison, WI). The plasmids pUNO1-hNOD1 and pUNO-hNOD2a for the expression of human NOD1 and NOD2, respectively, under a constitutive promoter, were purchased from Invivogen (San Diego, CA). pcDNA3.1 was from Life Technologies (Paisley, UK). The plasmid pcDNA3.3-Cas9n, encoding Cas9 nickase (Cas9n), was derived from the pcDNA3.3-Cas9 plasmid (Addgene #41815) [28] by introducing a D(10)A mutation into Cas9 coding sequence. The pKS-gRNA-BB vector, designed for custom sgRNA expression, was generated as described [29]. pKS-gRNA-BB contains a U6 RNA polymerase III promoter required for sgRNA transcription, two BbsI restriction sites for cloning of a synthetic double-stranded oligodeoxynucleotide (dsODN) encoding the gene-specific part of sgRNA, and a sequence encoding the constant part of sgRNA [29].

sgRNA target sites in the 5’-most regions of the *NOD1* and *NOD2* coding sequences were selected according to the published rules [27] using an online tool at http://crispr.mit.edu/. Since Cas9n cuts only one DNA strand, each gene was targeted by a pair of sgRNAs, which bind opposite DNA strands at a distance of less than 20 bp [27]. Single-stranded, 5’-phosphorylated ODNs corresponding to the gene-specific parts of sgRNAs (S1 Table) were synthesized at Syntol (Moscow, Russia). Pairs of complementary ODNs were annealed to yield dsODNs with 4-nucleotide 5’-overhangs. The pKS-gRNA-BB vector was digested with a BbsI isoschizomer (BstV2I, Sibenzyme, Moscow, Russia) to create sticky ends compatible with the 5’-overhangs of the dsODNs. The vector and dsODN inserts were ligated, and the inserts were verified by sequencing.

### Muropeptides and other reagents

Muropeptide compounds used are shown in Fig 1. The muropeptides GM-triDAP, GM-tetraDAP and diGM-tetraDAP were isolated from *S*. *typhi* as described earlier [19]. Briefly, peptidoglycan of *S*. *typhi* was rigorously purified by repetitive phenol extractions and subjected to an overnight lysozyme treatment, whereafter the low-molecular-weight fraction (<5 kDa) containing muropeptides was separated by dialysis. GM-triDAP, GM-tetraDAP and diGM-tetraDAP were isolated from the dialysate by HPLC. Chemical identity and purity of each muropeptide were confirmed by electrospray ionization time-of-flight (ESI-TOF) mass spectrometry (S1 Fig) using a micrOTOF II instrument (Bruker Daltonics, Billerica, MA). Reduced GM-triDAP (GM-triDAP-r) and GM-tetraDAP (GM-tetraDAP-r) were obtained by treating native muropeptides with NaBH_4_ under alkaline conditions as described [30], with subsequent purification by HPLC.

Isoglutamyl-*meso*-diaminopimelic acid (iE-DAP) and muramyl dipeptide (MDP) were purchased from Invivogen. Lipofectamine 2000 was from Life Technologies. A broad-range protease inhibitor cocktail (PIC) was from Sigma (St. Louis, MO; cat. # P8340) and contained 104 mM 4-(2-aminoethyl)benzenesulfonyl fluoride hydrochloride, 0.08 mM aprotinin, 2 mM leupeptin, 4 mM bestatin, 1.5 mM leupeptin A and 1.4 mM E-64.

### Generation of modified HEK293T cells

The HEK293T cell line was obtained from ATCC and maintained in DMEM supplemented with 2 mM L-glutamine and 10% heat-inactivated fetal calf serum (PAA, Pasching, Austria).

To generate a cell line where the luc2P gene under an NF-κB-dependent promoter would be stably integrated into the genome, HEK293T cells were lipofected with pGL4.32[luc2P/NF-kB-RE/Hygro] and selected for 1 month in the presence of 200 μg/ml hygromycin B (Life Technologies). The resulting 293Luc cell line was maintained in hygromycin B and regularly checked for the presence of the transgene by assessing luc2P expression induced by recombinant human tumor necrosis factor (TNF; Life Technologies).

To generate single-gene KO cells using the CRISPR-Cas9 technology, the 293Luc cells were seeded in 24-well plates and cotransfected with three plasmid constructs: two constructs (250 ng each) encoding sgRNAs targeting the gene of interest, and 500 ng of pcDNA3.3-Cas9n. Fourty-eight hours later, cells were re-seeded in 96-well plates at a mean of one cell per well. After approximately two weeks, wells containing single colonies were trypsinized, and clones were tested for target gene KO. A *NOD* gene was considered knocked-out if no NF-κB-dependent luciferase response (stimulation index less than 2) was observed upon stimulation with the smallest specific agonist (iE-DAP at 300 μM in case of NOD1, MDP at 1 μM in case of NOD2 [15]). Eligible clones were further checked for the absence of off-target effects, using following criteria: (i) no gross change of response to the specific agonist of the untargeted NOD receptor; (ii) no gross change of response to TNF; (iii) growth rate comparable to that of the parental 293Luc cell line. In selected clones matching these criteria, targeted genomic loci were PCR-amplified using Pfu DNA polymerase, cloned into pJet1.2/blunt vector (Fermentas, Vilnius, Lithuania), and sequenced. For each locus and cell clone, 6–8 plasmid clones were analysed. Following primers were used to amplify the *NOD1* and *NOD2* target sites: NOD1 forward 5’-GAGAGGACACACGCAGCTGAAG-3’; NOD1 reverse 5’-ACTGTGCAACCTGCTAACCC-3’; NOD2 forward 5’-TGGAAGGCTTCGAGAGTGTC-3’; NOD2 reverse 5’-TAGGGGAAATCCCATGGACC-3’.

To create cells with a double *NOD1/NOD2* KO, 293Luc cells with a single *NOD1* KO were subjected to the *NOD2* KO procedure described above; similar selection criteria were applied, except that the double KO cells should have responded to neither iE-DAP nor MDP.

### Cell stimulation and measurement of chemiluminescence

In model 1, 293Luc cells and their KO derivatives were seeded in 96-well flat-bottom plates at 10^4^ cells per 100 μl per well. Fourty-eight hrs later, when the cells were 70–80% confluent, muropeptides or recombinant human TNF (100 ng/ml; Life Technologies) were added in duplicates or triplicates. Negative control wells received an equal volume of DMEM. PIC, if used, was added 15 min. prior to addition of stimuli at a 1/1000 dilution, which was determined to be non-toxic for cells. After another 24 hrs, BrightGlow® substrate solution (Promega) was added to all wells, and chemiluminescence (relative luminescence units [RLU[) was measured by a plate chemiluminometer Lucy II (Anthos, Austria). To adjust for inter-experimental variability, responses to muropeptides were expressed as percents of response of the given cell line to TNF in the given experiment.

In model 2, 293Luc cells cultured as above were treated, in duplicates, with muropeptides together with pUNO1-hNOD1 (0.08 ng/well) or pUNO-hNOD2a (2 ng/well) or pcDNA3.1 (negative control, 2 ng/well) in the presence of Lipofectamine 2000 (0.4 μl/well). The doses of plasmids were determined in preliminary titration experiments as those not causing substantial NF-κB activation in the absence of muropeptide ligands (S2 Fig). NF-κB-dependent luc2P expression was measured 24 hrs later as described above. Since NF-κB activation in this model can be caused by NOD1/2 expression vectors alone and/or by activation of endogenous NOD1/2, results were expressed as indices of synergy (IS): IS = A / (B + C), where A, B and C are RLU of cells transfected with (A) the NOD expression plasmid plus the muropeptide at the given concentration, (B) the NOD expression plasmid only and (C) the muropeptide at the given concentration plus the control plasmid.

### Culture and stimulation of monocytes and macrophages

Heparinized venous blood was obtained from healthy donors. Blood sampling was approved by the local Ethical Committee of the National Research Center “Institute of Immunology” (Protocol 12/2015), and written informed consent was obtained from all donors. PBMC were isolated by the routine Ficoll density gradient centrifugation. After three washes, PBMC were resuspended in RPMI supplemented with 1% human AB serum (both from PAA) at 10^7^ cells per ml.

For monocyte stimulation experiments, PBMC were plated in 96-well flat-bottomed plates at 6x10^5^ cells/well and incubated for 1 hr at 37°C in 5% CO_2_. Non-adherent cells were removed by two washes with warm RPMI, and wells were filled with complete culture medium (CCM), which was RPMI supplemented with 2 mM L-Glutamine and 10% fetal calf serum. Immediately thereafter, muropeptides or LPS *E*.*coli* O111:B4 (Sigma) were added in duplicates, and cell culture supernatants were harvested 24 hrs later.

To generate macrophages, monocytes were isolated as above (except that 6-well plates were used), and cultured for 6 days in CCM supplemented with 40 ng/ml recombinant human granulocyte-macrophage colony-stimulating factor (GM-CSF; Life Technologies) [19]. Medium was refreshed on day 3. On day 6, macrophages were trypsinized, counted, and replated in 96-well plates in CCM at 4x10^4^/well. Cells were allowed to attach and stimulated, in duplicates, with muropeptides or LPS for 24 hrs, whereafter supernatants were collected.

### siRNA-mediated knock-down of NOD1 and NOD2 in macrophages

Sequences of short interfering RNAs (siRNA) were as follows [31]: NOD1 sense, 5’-GGGUGAGACCAUCUUCAUC*TT*-3’, NOD1 antisense, 5’-GAUGAAGAUGGUCUCACCC*TG*-3’, NOD2 sense, 5’-GGAAUUACCAGUCCCAUUG*TT*-3’, NOD2 antisense, 5’-CAAUGGGACUGGUAAUUCC*TG*-3’, scrambled sense, 5’-UUCUCCGAACGUGUCACGU*TT*-3’, scrambled antisense, 5’-ACGUGACACGUUCGGAGAA*TT*-3’, where 3’ deoxyribonucleotide overhangs are italicized. To obtain fluorescein (FAM)-labeled siRNA, the scrambled antisense oligo was 3’-labeled with FAM. Single-stranded oligos were ordered from Syntol and diluted in 100 mM NaCl, 50 mM HEPES (pH 7.4). To obtain siRNA duplexes, pairs of complementary oligos were mixed at final concentrations of 20 μM, heated at 90°C for 3 min and slowly cooled to room temperature.

All transfection conditions were established in extensive preliminary experiments (see below). The 6-day macrophages generated as above were plated in 96-well plates at 10^4^ cells/well in CCM and allowed to attach. Complexes consisting of 8 pmol/well siRNA and 0.4 μl/well Lipofectamine 2000 (Life Technologies) were added for 15 hrs, whereafter medium was replaced with fresh CCM supplemented with 20 ng/ml GM-CSF. Cells were cultured for another 45 hrs (altogether 60 hrs post transfection), at which point muropeptides were added, and supernatants were collected 12 hrs later.

### ELISA

Levels of TNF in the supernatants were analysed by sandwich ELISA using a reagent kit from Cytokine (St-Petersburg, Russia).

### Measurement of NOD1, NOD2 and TLR4 expression by RT-PCR

Total RNA from monolayer 293Luc cells, monocytes and macrophages was extracted by TRI Reagent (Sigma) as recommended by the manufacturer. 0,5 μg RNA was reverse-transcribed using RevertAid kit (Fermentas). Amplifications were done in a 7300 Real-Time PCR System (Applied Biosystems, Foster City, CA) in duplicates using the standard Taqman protocol. All primer/probe kits were from Applied Biosystems (assay IDs: human *NOD1*, Hs00196075_m1; human *NOD2*, Hs00223394_m1; human *TLR4*, Hs00152932_m1; human ACTB, 4333762F). Relative NOD1, NOD2 and TLR4 mRNA expression was calculated by the 2^−ΔCt^ method using ACTB (β-actin) as the house-keeping gene.

### Immunostaining and flow cytometry

293Luc cells were trypsinized, fixed with 4% paraformaldehyde (Sigma) for 20 min at +4°C, washed with PBS and permeabilized by PBS containing 0.1% saponin (Sigma) and 0.5% BSA (wash buffer). Permeabilized cells were resuspended in the wash buffer and incubated with rabbit anti-human NOD1 polyclonal or mouse anti-human NOD2 monoclonal (2D9) antibodies (both from Santa Cruz Biotechnologies; cat. # sc-99163 and sc-56168, respectively) for 30 min. at room temperature. After two washes, cells were stained with secondary donkey anti-rabbit IgG NorthernLights^TM^ NL493-labelled antibodies (R&D Systems, Minneapolis, MN) or with goat anti-mouse IgG Alexa Fluor 488-labelled antibodies (Thermo Fischer Scientific, Waltham, MA) for 30 min. at room temperature. Cells were washed once with the wash buffer and once with 0.5% BSA in PBS, resuspended in the latter buffer and analysed using a Cytomics FC500 flow cytometer equipped with CXP software (both from Beckman Coulter).

### Statistics

All data are presented as mean ± s.d. Groups were compared by Student’s t-test.

## Results

### Endogenous expression of NOD1 and NOD2 in HEK293T cells

To reduce the number of plasmid constructs transfected into model cells, we generated a variant of HEK293T cells, named 293Luc, wherein the NF-κB-luc2P transgene is stably integrated into the genome. To confirm the integration, 293Luc cells were treated with TNF in each experiment, which always triggered a strong luciferase response (15329 ± 2865 RLU) with a low background signal (11.2 ± 6.7 RLU) and a stimulation index of 1740 ± 931 (S3A Fig).

**Fig 2:**
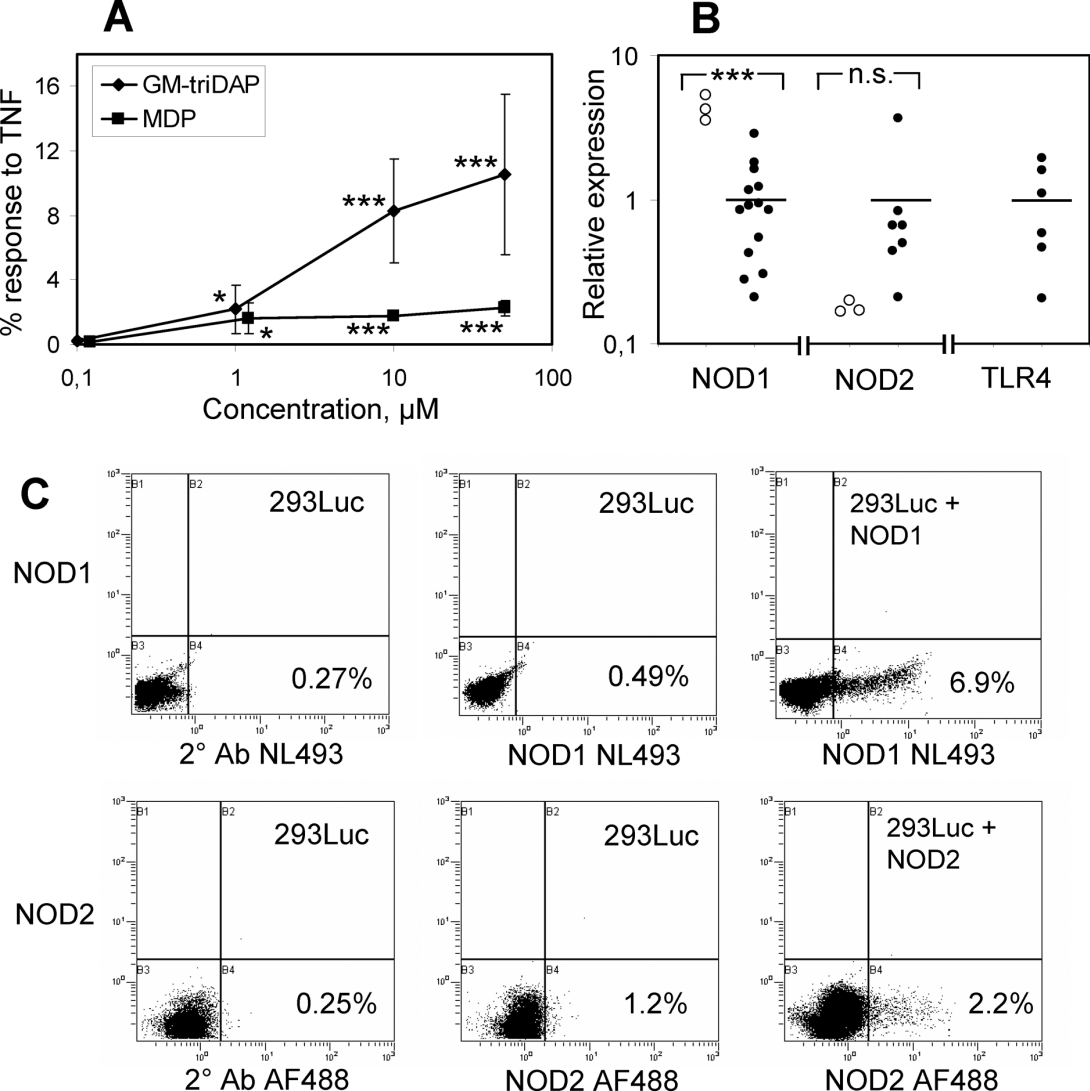
Endogenous expression of NOD1 and NOD2 by the 293Luc cells. (A) 293Luc cells were stimulated with GM-triDAP or MDP for 24 hrs, whereafter luc2P activity was measured as described in Materials and Methods. Mean ± s.d. of 5 independent experiments. * p < 0.05, *** p < 0.001 compared to unstimulated cells. (B) Relative expression of *NOD1*, *NOD2* and *TLR4* mRNA in 293Luc cells (open circles; three independent measurements) and in different monocyte-derived macrophage preparations (closed circles) as measured by RT-PCR. Horizontal lines denote mean mRNA expression in macrophages (adjusted such that it equals 1). *** p < 0.001 by Student’s t-test. TLR4 mRNA was below the level of detection in 293Luc cells. (C) Expression of NOD1 and NOD2 proteins measured by flow cytometry in intact 293Luc cells (middle plots) or 24 hrs after transfection with NOD1 or NOD2 expression plasmids (right plots). Left plots, staining controls (omission of primary antibodies). Both plasmids were used at 2.5 μg per a subconfluent well of a 6-well plate. In all dot plots, percentages of positive cells are indicated; shown is one experiment out of 2 with similar results. NL, Northern lights; AF, Alexa Fluor.

When 293Luc cells were stimulated with NOD1 (GM-triDAP) or NOD2 (MDP) agonists, without concomitant NOD1 or NOD2 transfection, NF-κB activation was consistently observed at agonist concentrations of 1 μM and higher (Fig 2A), suggesting endogenous expression of NOD1 and NOD2. RT-PCR showed that both *NOD1* and *NOD2* mRNAs were expressed in 293Luc cells (Fig 2B), roughly at the same order of magnitude as in macrophages, a cell type known to robustly respond to NOD1 and NOD2 agonists [19]. Thus, *NOD1* expression in 293Luc was 4.3 times higher, and *NOD2* expression 5.6 times lower than the average expressions in macrophages (Fig 2B). As a control, *TLR4* mRNA was detected in macrophages and not in 293Luc cells (Fig 2B). Despite these functional and mRNA data, endogenous NOD1 and NOD2 proteins could not be reliably detected in 293Luc cells, although transgenic, plasmid-encoded NOD proteins were readily detectable in a subset of cells (Fig 2C).

### Generation of NOD1- and NOD2-KO cells

We took advantage of endogenous NOD1 and NOD2 expression in 293Luc cells to create a novel model to assess agonistic properties of muropeptides. CRISPR-Cas9 technology was used to knock out *NOD1* or *NOD2* or both genes in 293Luc cells, in order to obtain cells expressing NOD2 only, NOD1 only, or none of them. First, single-gene KO of *NOD1* or *NOD2* were performed. The frequency of putative KO clones, i.e. those not responding to smallest-size NOD1 or NOD2 agonists (iE-DAP and MDP, respectively), was 8 out of the 42 tested (19%) in the case of NOD1, and 8 out of 29 (27.6%) in the case of NOD2 (S3B Fig, S3C Fig). After checking for off-target effects, we selected one *NOD1*-KO and one *NOD2*-KO clone, which were named 293Luc-ΔNOD1 and 293Luc-ΔNOD2. To create the double-KO 293Luc-ΔNOD1/2 cells, *NOD2* KO was carried out in the 293Luc-ΔNOD1 cells, and one clone was selected. Responses of the selected clones to iE-DAP, MDP and TNF, confirming the specificity of KO and absence of gross off-target effects, are shown in Table 1.

**Table 1:** Chemiluminescence of parental and NOD1/NOD2-KO 293Luc cells treated with smallest NOD1/NOD2 agonists or TNF for 24 hrs (RLU, mean±s.d. of triplicates)*.

	293Luc(parental)	293Luc-ΔNOD1	293Luc-ΔNOD2	293Luc-ΔNOD1/2
No stimulation	1.9±1.4	3.5±1.1	1.4±0.4	1.4±0.3
iE-DAP (300 μM)	73.5±6.4	3.4±0.2	60.5±16.3	1.2±0.4
MDP (1 μM)	113±24	132±12	0.45±0.2	1.1±0.1
TNF (100 ng/ml)	9425±91	7141±990	10459±584	4576±115

* Shown is one experiment out of 2 with similar results.

*NOD1* and *NOD2* KO in the three clones selected were verified by sequencing genomic target sites. In 293Luc-ΔNOD1 cells, two mutated *NOD1* alleles were identified: allele 1 contained a 34-bp deletion, and allele 2 contained two insertions of 90 bp and 7 bp (S4A Fig). Both the deletion and the insertions created frameshifts, leading to altered amino acid sequences downstream of mutation sites and to premature stop codons. In 293Luc-ΔNOD2 cells, two mutated alleles with 21-bp and 51-bp deletions were found (S4B Fig). Despite no frameshifts, the deletions probably inactivated the NOD2 protein, since no response to the specific NOD2 agonist was observed (Table 1). In 293Luc-ΔNOD1/2 cells, three mutated alleles of the *NOD2* gene (all with deletions) were identified (S4B Fig), possibly because the original HEK293T cells are variably hypotriploid, and some cells may contain three copies of *NOD2* gene. Two of the deletions created frameshifts and premature stop codons (S4B Fig).

### NOD1 and NOD2 activation by meso-DAP-containing muropeptides

Using the generated KO cells, we examined several muropeptides from Gram-negative bacteria for the ability to activate NOD1 and NOD2. The parental 293Luc cell line responded not only to the ‘classical’ NOD1 and NOD2 agonists, such as GM-triDAP and MDP, but also to GM-tetraDAP and diGM-tetraDAP (Table 2), which contain meso-DAP in a non-terminal position. Responses of 293Luc cells to GM-tetraDAP and diGM-tetraDAP were weaker than responses to GM-triDAP and were observed at concentrations of 10 μM and higher. At 50 μM, the magnitude of responses of 293Luc cells to GM-tetraDAP and diGM-tetraDAP was comparable with that to MDP (Table 2).

**Table 2:** Responses of 293Luc, 293Luc-ΔNOD1, 293Luc-ΔNOD2 and 293Luc-ΔNOD1/2 cells to muropeptides (% response to TNF). Luc2P activity was measured 24 hrs after addition of muropeptides. Mean ± s.d. of 3 to 9 independent experiments per data point.

Stimulus	Concentration (μM)	293Luc	293Luc-ΔNOD1	293Luc-ΔNOD2	293Luc-ΔNOD1/2
None	–	0	0	0	0
iE-DAP	50	0.13±0.06	n.d.	n.d.	n.d.
	300	2.3±2.2	0	1.6±1.4	0
MDP	0.1	0.15±0.17	0.2±0.15	n.d.	n.d.
	1	1.6±0.96*	1.8±0.8*	0.06±0.09	0.01±0.03
	10	1.8±0.31***	2.1±0.67***	0.05±0.1	0.01±0.01
	50	2.3±0.49***	2.6±0.86**	0.06±0.08	0
GM-triDAP	0.1	0.21±0.18	0	0.44±0.2	n.d.
	1	2.2±1.5*	0.06±0.01	2.7±0.18*	n.d.
	10	8.3±3.2***	0.04±0.05	9.4±4.5**	0
	50	10.5±5.0***	0.43±0.34**	13.6±4.1***	0
GM-tetraDAP	1	0.02±0.04	0	0.04±0.02	n.d.
	10	0.67±0.64*	0.22±0.05*	0.36±0.22*	0
	50	3.1±1.3***	1.0±0.32***	3.8±2.7*	0
diGM-tetraDAP	1	0.1±0.1	0.08±0.15	0.13±0.12	n.d.
	10	0.4±0.23*	0	0.41±0.32*	0
	50	2.4±1.1**	1.1±0.69**	1.7±1.3*	0
TNF	100 ng/ml	100	100	100	100

* p < 0.05

** p < 0.01

*** p < 0.001 compared to 0% (unstimulated cells of the same cell line). N.d., not done.

In 293Luc-ΔNOD1 cells, where only NOD2 is functional, responses to MDP were not affected, while responses to GM-triDAP were strongly diminished, but a minor response was still detectable at 50 μM (Table 2). Furthermore, 293Luc-ΔNOD1 cells responded to GM-tetraDAP at 10 and 50 μM, and to diGM-tetraDAP at 50 μM, magnitudes of these responses being around 30% of those seen in 293Luc cells (Table 2).

In 293Luc-ΔNOD2 cells, responses to GM-triDAP, GM-tetraDAP and diGM-tetraDAP were similar to those in 293Luc cells, while response to MDP was absent at all concentrations tested (Table 2). 293Luc-ΔNOD1/2 cells did not respond to any muropeptide tested (Table 2), confirming the role of NOD2 in the recognition of meso-DAP-containing muropeptides and ruling out the involvement of any other receptor in muropeptide recognition in this model. We concluded that in the 293Luc cells, GM-triDAP is recognized through NOD1 with a minor contribution from NOD2, whereas GM-tetraDAP and diGM-tetraDAP are recognized through both receptors to a comparable extent, at least in this experimental system.

**Fig 3:**
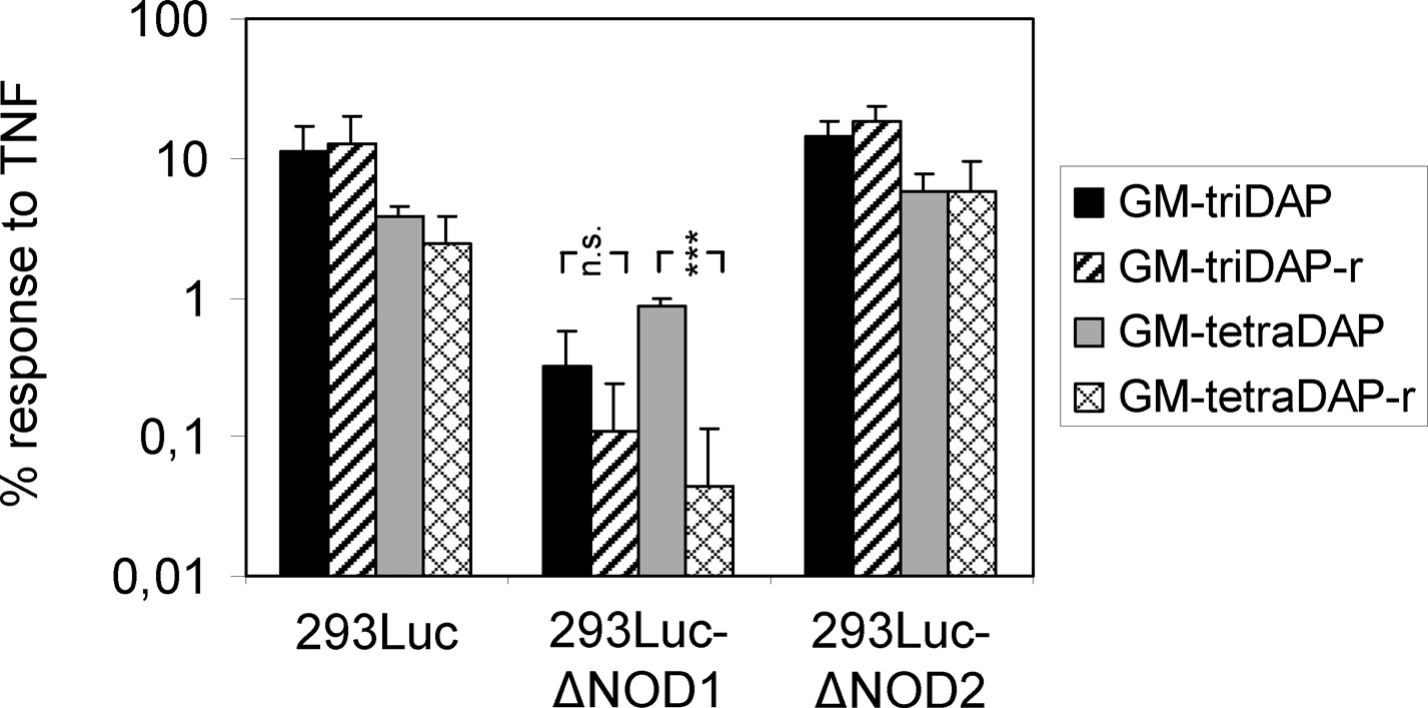
The role of NOD2 in the recognition of meso-DAP-containing muropeptides. 293Luc, 293Luc-ΔNOD1 and 293Luc-ΔNOD2 cells were stimulated with native GM-triDAP or GM-tetraDAP or with their reduced derivatives (GM-triDAP-r, GM-tetraDAP-r), each at 50 μM, and luc2P activity was measured 24 hrs later. Mean ± s.d. of 5 independent experiments. *** p < 0.001.

To corroborate the role of NOD2 in the recognition of meso-DAP-containing muropeptides, additional experiments were done. First, using mass-spectrometry, we verified that MDP or GMDP (i.e., classical NOD2 agonists) were absent from our GM-triDAP, GM-tetraDAP and diGM-tetraDAP preparations (S1 Fig). Second, GM-triDAP and GM-tetraDAP were chemically reduced in order to destroy the pyranose ring in the MurNAc residue, which is critical for NOD2 recognition and dispensable for NOD1 recognition [15]. The reduced GM-tetraDAP retained its activity in 293Luc-ΔNOD2 cells, expressing NOD1 only, but lost activity in 293Luc-ΔNOD1 cells, expressing NOD2 only (Fig 3), which supports the role for NOD2 in GM-tetraDAP recognition. Response to native GM-triDAP in 293Luc-ΔNOD1 cells was initially low and was further diminished by chemical reduction of the agonist, though not statistically significant (Fig 3).

**Fig 4:**
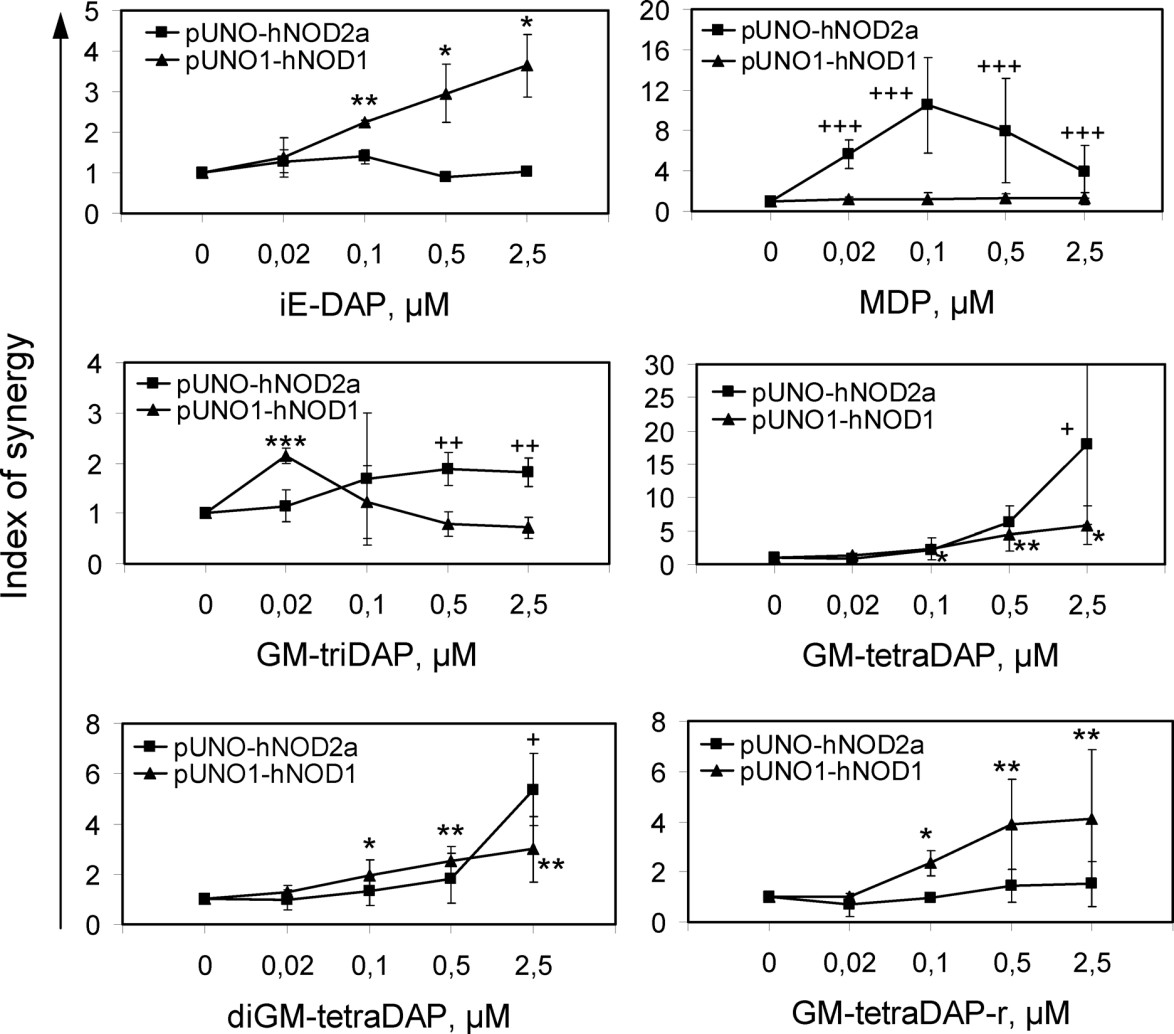
Activation of NOD1 and NOD2 by muropeptides in a NOD1/NOD2 overexpression model. 293Luc cells were lipofected with NOD1 or NOD2 expression plasmids together with the muropeptides at indicated concentrations, as described in Materials and Methods, and luc2P activity was measured 24 hrs later. Results are shown as indices of synergy. Mean ± s.d. of 3 to 5 independent experiments per data point. * p < 0.05, ** p < 0.01, *** p < 0.001 for NOD1-transfected cells, + p < 0.05, ++ p < 0.01, +++ p < 0.001 for NOD2-transfected cells compared to baseline (plasmids without muropeptides).

Third, we also used the standard approach and overexpressed NOD1 and NOD2 in 293Luc cells using plasmid constructs. NOD2 overexpression significantly augmented responses to MDP, GM-tetraDAP, diGM-tetraDAP, and to some extent to GM-triDAP as well, but not to GM-tetraDAP-r and iE-DAP (Fig 4 and S5 Fig). These data are consistent with those obtained in 293Luc-ΔNOD1 cells (Table 2) and confirm the role of NOD2 in the recognition of meso-DAP-containing muropeptides. In Fig 4 and S5 Fig, muropeptides were delivered intracellularly together with the plasmids, hence lower concentrations of muropeptides were sufficient to activate NOD1 and NOD2 as compared to experiments shown in Table 2 or Fig 2A, where muropeptides were simply added to the medium. It should be noted that, irrespectively of the muropeptide delivery mode, concentrations of meso-DAP-containing muropeptides required to achieve a statistically significant NOD2 activation were 1–2 orders of magnitude higher than those of MDP.

NOD1 overexpression significantly augmented responses to GM-tetraDAP, diGM-tetraDAP (albeit to a lesser extent than NOD2 overexpression) as well as to iE-DAP (Fig 4), which is in a full agreement with data in NOD2-KO cells. Interestingly, responses of NOD1-transfected cells to GM-triDAP, if expressed as indices of synergy, were significantly increased only at the agonist concentration of 0.02 μM (Fig 4), because at higher agonist concentrations, NOD1-transfected and control-transfected cells yielded similar RLU values (S5 Fig). Presumably, the NOD1-agonistic properties of GM-triDAP are so strong that endogenous NOD1 levels are sufficient to induce full-scale NF-κB activation.

### The pre-processing requirements for NOD1 and NOD2 agonists

**Fig 5:**
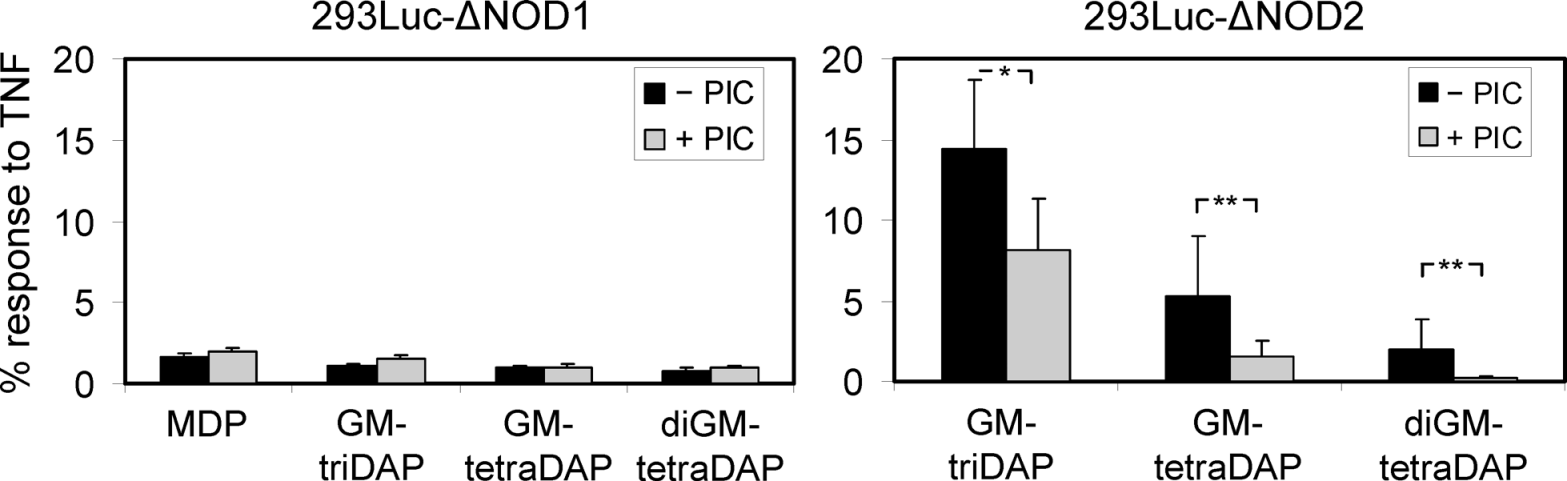
The effect of a protease inhibitor cocktail (PIC) on the recognition of muropeptides by NOD1 and NOD2. 293Luc-ΔNOD1 and 293Luc-ΔNOD2 cells were pre-incubated without or with PIC (1/1000) for 15 min., then stimulated with MDP, GM-triDAP, GM-tetraDAP or diGM-tetraDAP (all at 50 μM) for 24 hrs. Mean ± s.d. of 4 independent experiments. * p < 0.05, ** p < 0.01.

We asked whether GM-tetraDAP and diGM-tetraDAP can be recognized by NOD1 or NOD2 directly, or should be pre-processed in endosomes to remove ‘unnecessary’ amino-acid residues. To this end, 293Luc-ΔNOD1 and 293Luc-ΔNOD2 cells were stimulated with muropeptides in the presence of PIC taken at a low, non-toxic concentration. In 293Luc-ΔNOD1 cells, PIC did not influence muropeptide-induced NF-κB activation (Fig 5), implying that NOD2 can recognize GM-triDAP, GM-tetraDAP and diGM-tetraDAP in their native forms. In 293Luc-ΔNOD2 cells, PIC decreased the muropeptide-triggered NF-κB activation (Fig 5). The response to GM-triDAP was reduced only by 1.5 fold, whereas responses to GM-tetraDAP and diGM-tetraDAP were decreased by 3.4 and 10.2 fold, respectively (in the latter case, almost to the baseline) (Fig 5). We conclude that ‘unmasking’ of the meso-DAP residue by peptidases is probably required for GM-tetraDAP and diGM-tetraDAP to activate NOD1.

### The role of NOD1 and NOD2 in muropeptide recognition in monocytes and macrophages

**Fig 6:**
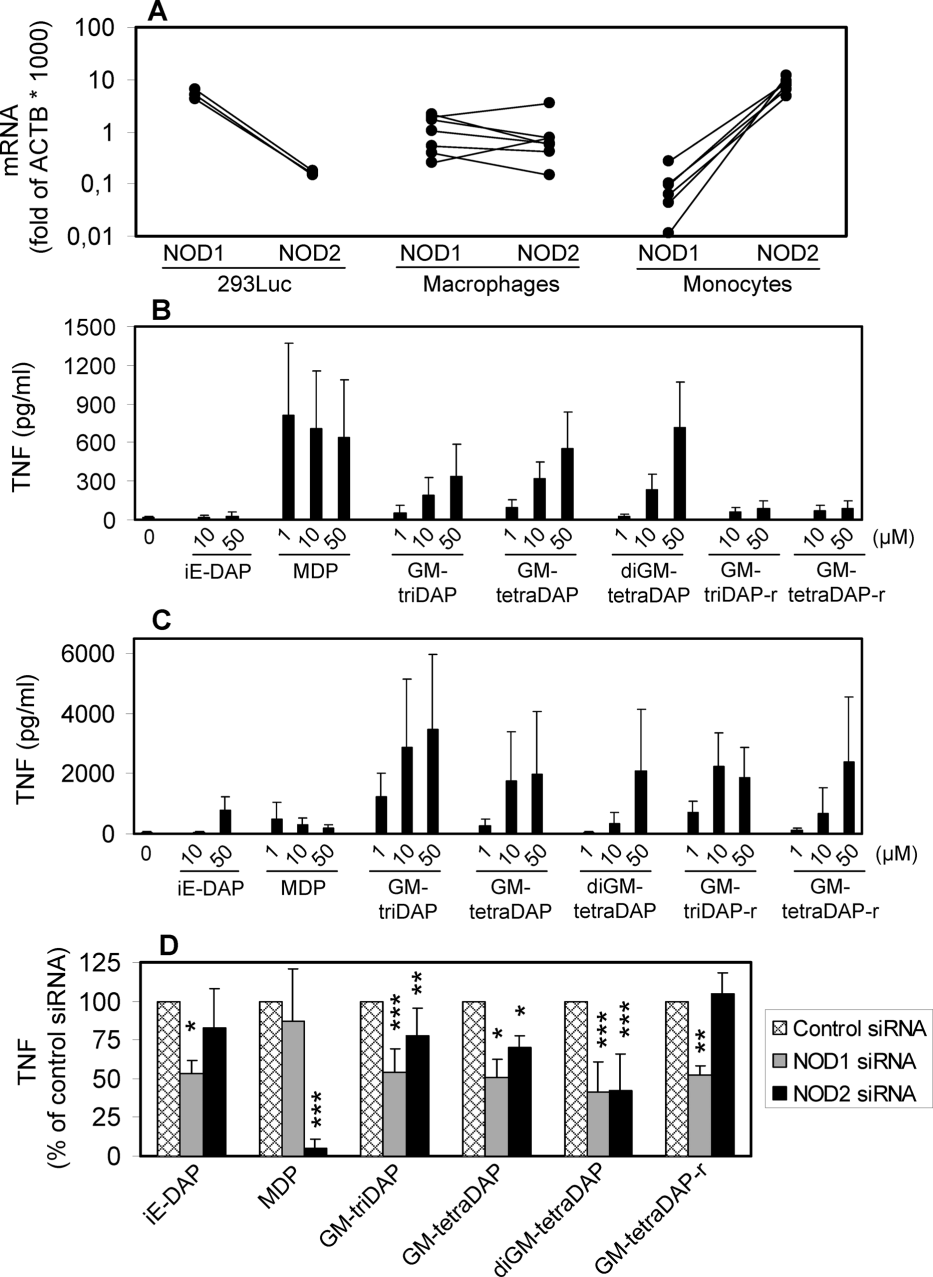
The role of NOD1 and NOD2 in muropeptide recognition in monocytes and macrophages. A, relative expression of NOD1 and NOD2 mRNA in 293Luc cells (3 independent measurements), macrophages (7 donors) and monocytes (6 donors), expressed as fold of ACTB mRNA in the same samples (RT-PCR). B and C, blood monocytes from 5 independent donors (B) or monocyte-derived macrophages from 10 independent donors (C) were stimulated with muropeptides, and levels of TNF in the supernatants were assessed 24 hrs later. D, macrophages were transfected with siRNA and stimulated with muropeptides, and levels of TNF in the supernatants were measured by ELISA (see Materials and Methods for detail). All muropeptides were used at 50 μM, except GM-triDAP (10 μM) and MDP (2 μM). Numbers of independent experiments were 10 for MDP and GM-triDAP, 7 for diGM-tetraDAP, and 3 for iE-DAP, GM-tetraDAP and GM-tetraDAP-r. * p < 0.05, ** p < 0.01, *** p < 0.001 compared to stimulation with the same muropeptide in the presence of control siRNA. In B, C, D, all data are Mean ± s.d.

The low NOD2 expression in 293Luc cells (Fig 2B) may result in underestimation of NOD2-specific responses. We therefore searched for cells with other NOD1/NOD2 ratios. Literature suggests that in blood monocytes, expression of NOD2 may prevail over that of NOD1 [23, 24]. Indeed, the pattern of NOD1/NOD2 expression in monocytes was exactly the reverse of that in 293Luc cells (Fig 6A). NOD1 relative expression in monocytes was around 50 times lower, and NOD2 expression around 50 times higher than in 293Luc cells. In agreement with these expression data, monocytes robustly produced TNF in response to MDP, but did not respond to iE-DAP (Fig 6B). Responses to GM-triDAP, GM-tetraDAP and diGM-tetraDAP were of intermediate magnitude, and GM-triDAP was a weaker activator than the other two muropeptides, which is consistent with their acting via NOD2 rather than NOD1 (compare with Table 2). To see whether these responses were NOD1- or NOD2-mediated, we used reduced derivatives of GM-triDAP and GM-tetraDAP, which are recognized by NOD1 only (Fig 3 and [15]). Strikingly, reduction of the muropeptides resulted in a 70–80% loss of their activity in monocytes (Fig 6B). Thus, the effects of GM-triDAP, GM-tetraDAP and presumably diGM-tetraDAP in monocytes are mediated mainly through NOD2.

In monocyte-derived macrophages, intermediate and mutually balanced expression of NOD1 and NOD2 was observed (Fig 6A). Accordingly, macrophages did respond to iE-DAP (Fig 5C). In agreement with our previous report [19], and similarly to the situation in 293Luc cells (Table 2), GM-triDAP was the strongest inducer of TNF in macrophages, followed by GM-tetraDAP and diGM-tetraDAP (Fig 6C). Chemical reduction of GM-triDAP and GM-tetraDAP resulted in some decrease of their activity, which was not as striking as in monocytes, suggesting lesser contribution of NOD2 to their recognition in macrophages. Accordingly, siRNA-mediated knock-down of NOD1 (see S6 Fig for method establishment) caused a significant, around 50% reduction of responses to iE-DAP, GM-triDAP, GM-tetraDAP, diGM-tetraDAP and GM-tetraDAP-r (Fig 6D). Knock-down of NOD2 fully blocked responses to MDP, but had a smaller effect on GM-triDAP-, GM-tetraDAP- and diGM-tetraDAP-induced responses (Fig 6D). Collectively, these results confirm the data obtained in 293Luc cells and define the contribution of NOD1 and NOD2 in the recognition of meso-DAP-containing muropeptides in primary cells.

## Discussion

Muropeptides play an important role in the activation of innate immune responses upon bacterial infections, and are also viewed as promising immunostimulants and vaccine adjuvants. Two recent works have demonstrated that MDP and L-alanyl-γ-D-glutamyl-meso-DAP (triDAP) can directly bind NOD2 and NOD1, respectively [32, 33]. However, in most settings, conclusions about NOD1- and NOD2-agonistic properties are based upon functional studies, hence the need of a system allowing to specifically assess NOD1 and NOD2 activation.

The ‘standard’ experimental system used to test potential NOD1 and NOD2 agonists is based on plasmid-driven overexpression of the receptors in HEK293T cells. This system has several disadvantages: (1) it is necessary to transfect cells with plasmids in each experiment, which is time- and reagent-consuming; (2) transfection with NOD1 and NOD2 expression plasmids results in activation of NF-κB in the absence of muropeptides ([23, 24] and S2 Fig), which requires careful titration of plasmid doses; (3) the expression of transgenic NOD1 and NOD2 is heterogeneous, being unphysiologically high in some cells and absent in others (Fig 2C); (4) endogenous NOD1 and NOD2 activation interferes with the read-out (Fig 4). Another model is based on RNAi-mediated knock-down of endogenous *NOD1* or *NOD2* in primary or cultured human cells [31]. However, with this technology, it is hard to completely eliminate target protein expression, especially in primary cells. Murine *Nod1*- or *Nod2*-KO cells, expressing only one functioning Nod receptor, meet the specificity criterion, but cannot substitute for human cells because of inter-species differences in muropeptide recognition [18].

The novel model presented here, based upon KO of endogenous *NOD1* and/or *NOD2* genes in human HEK293T-derived cells, has following advantages: (1) a permanent KO of *NOD1* and/or *NOD2*, creating cells expressing only one functional NOD receptor, or none of them; (2) no need for plasmid transfection, and hence lack of non-specific NF-κB activation; (3) physiological levels of NOD1 and NOD2 expression; (4) no interference from endogenous receptor activation. A drawback of this model is the relatively low expression of *NOD2* as compared to *NOD1* (Fig 2B), which may lead to underestimation of NOD2-specific responses.

Using this novel system and several traditional approaches, we refine the role of NOD1 and NOD2 in muropeptide recognition. First, we show that muropeptides GM-tetraDAP and diGM-tetraDAP, where the meso-DAP residue is non-terminal, can activate NOD1 (Table 2, Fig 4). Both muropeptides surpass iE-DAP, but are inferior to the strongest known NOD1 agonist, GM-triDAP (Table 2). While NOD1 agonism of a GM-tetraDAP analogue (M-tetraDAP) has been reported [17], data on diGM-tetraDAP are novel. In order to activate NOD1, GM-tetraDAP and diGM-tetraDAP probably need to be ‘trimmed’ down to GM-triDAP by endosomal peptidases (Fig 5). Thus, the exposed meso-DAP residue appears to be a critical feature of NOD1 agonists. By contrast, the integrity of the carbohydrate part appears of less importance (Fig 3), as shown earlier [15]. Since another kind of pre-processing has been suggested for NOD1 agonists, namely removal of the carbohydrate residues [25], it can be proposed that all muropeptides capable of NOD1 activation have to be pre-processed to triDAP, which would then be the only true NOD1 agonist derived from natural PGs.

Second, we show that NOD2 can recognize different meso-DAP-containing muropeptides (Table 2, Fig 4), which have been viewed either as specific NOD1 agonists (GM-tetraDAP, GM-triDAP) or not as NOD agonists at all (diGM-tetraDAP). Thus, GM-triDAP, GM-tetraDAP and diGM-tetraDAP are in effect dual NOD1/NOD2 agonists. A pre-requisite to NOD2 recognition of meso-DAP-containing muropeptides is an intact pyranose ring in the MurNAc residue (Figs 3 and 4). The peptide chains in NOD2-agonistic muropeptides can be of different length and complexity, and their peptidase pre-processing is probably not required (Fig 5). However, excessive negative charge at the C-terminus appears to interfere with NOD2 recognition, since GM-triDAP was the least effective activator of NOD2. In all, these data show that NOD2 can recognize Gram-negative muropeptides and, therefore, plays an even broader role in bacterial recognition than currently thought.

Our data on NOD2 agonistic properties of GM-tetraDAP disagree to some extent with an article by Wolfert et al [17], who reported no NOD2 activation by M-tetraDAP. An explanation to this discrepancy might be that Wolfert et al used M-tetraDAP at 5 μM without liposomes, whereas we used GM-tetraDAP at up to 50 μM without liposomes (Table 2, Figs 3, 5 and 6) or at up to 2.5 μM with liposomes (Fig 4), which, in both cases, may have resulted in higher cytosolic concentrations of GM-tetraDAP.

Innate immune recognition of GM-tetraDAP and diGM-tetraDAP is important, because, unlike GM-triDAP, these muropeptides are main break-down products of Gram-negative PGs [8, 34]. The ability of GM-tetraDAP and diGM-tetraDAP to activate innate immunity can be inferred from early works by Lederer’s group, who demonstrated comparable adjuvanticity of GM-tetraDAP, diGM-tetraDAP, GM-triDAP and MDP [16, 34]. At least in monocytes, GM-tetraDAP and diGM-tetraDAP induce an even stronger TNF production than does GM-triDAP (Fig 6B). In order to activate NOD1 and NOD2, GM-tetraDAP and diGM-tetraDAP should be present in culture media at relatively high concentrations not explored up to now (10–50 μM; Table 2). Nonetheless, these concentrations are relevant, because the reported Kd of the NOD1:triDAP complex (34.5 μM) falls exactly in this range [33]. In addition, high cytosolic concentrations of muropeptides can be reached when much smaller quantities of extracellular muropeptides are delivered to the cytosol using liposomes, or when the cell is infected with a cytosolic bacterium.

The mode of recognition of meso-DAP-containing muropeptides depends on the relative abundance of NOD1 and NOD2 in the given cell type. For example, in monocytes, where NOD2 predominates, these muropeptides are recognized primarily through NOD2 (Fig 6A and 6B). This may explain, at least in part, the reduced response to these ‘NOD1 agonists’ by PBMC from Crohn’s patients with the NOD2 frameshift mutation [20]. If the NOD1/NOD2 ratio is skewed towards NOD1, like in macrophages or 293Luc cells, the contribution of NOD1 to muropeptide recognition is more substantial (Table 2, Fig 6C).

Our data also broaden the range of theurapeutic applications of muropeptides. Depending on the clinical situation, one may consider administration of pure NOD1 agonists, such as reduced muropeptides, to preferentially stimulate local immunity, pure NOD2 agonists to achieve monocyte activation, or dual NOD1/NOD2 agonists, such as GM-triDAP, GM-tetraDAP or diGM-tetraDAP, to achieve a mixed effect.

In conclusion, we show that meso-DAP-containing muropeptides from Gram-negative bacteria have dual NOD1/NOD2-agonistic properties, which should be taken into account when assessing their biological activities and designing muropeptide-based immunostimulants or adjuvants.

## Supporting Information

S1 Fig Electrospray ionization time-of-flight (ESI-TOF) mass spectrometry of GM-triDAP, GM-tetraDAP and diGM-tetraDAP preparations. Each muropeptide is represented by a major peak corresponding to the native molecule and minor peaks corresponding to fragments devoid of one or more GlcNAc residues (these being GlcNAc residues per se or parts of MurNAc residues). Measured (calculated) molecular masses for [M–H]^−^ions are as follows: GM-triDAP native, 867.35 (867.56); GM-triDAP–GlcNAc, 664.27 (664.48); GM-triDAP– 2 GlcNAc, 461.19 (461.39); GM-tetraDAP native, 938.39 (938.64); GM-tetraDAP–GlcNAc, 735.31 (735.57); GM-tetraDAP– 2 GlcNAc 532.23 (532.5); diGM-tetraDAP native, 1859.68 (1859.25); diGM-tetraDAP– 2 GlcNAc, 1453.54 (1453.12), diGM-tetraDAP– 4 GlcNAc, 1047.4 (1047.97). Small peaks adjacent to some major peaks correspond to [M–2H+Na]^−^and [M–3H+2Na]^−^ions. In all three preps, no peaks corresponding to MDP ([M–H]^–^, 492.3) or glucosaminyl muramyl dipeptide (GMDP, [M–H]^–^, 695.5) were detected.
(TIF)

S2 Fig Transfection with NOD1 and NOD2 expression plasmids triggers NF-κB activation in the absence of muropeptide ligands. 293Luc cells were seeded in 96-well plates and transfected with the indicated doses of pUNO1-hNOD1, pUNO-hNOD2a or pcDNA3.1, and luc2P activity was measured 24 hrs later. Mean ± s.d., n = 3.
(TIF)

S3 Fig Generation of 293Luc, 293Luc-ΔNOD1 and 293Luc-ΔNOD2 cells. A, luc2P activity in 293Luc cells stimulated by TNF (100 ng/ml) for 24 hrs in 20 independent experiments performed over a two-year period. Shown are means of duplicates. B and C, 293Luc-derived clones with the putative NOD1 (B) or NOD2 (C) gene knock-out were stimulated by iE-DAP at 300 μM (B) or MDP at 1 μM, and luc2P activity was measured in 24 hrs. Non-responding clones (stimulation index less than 2) are marked by arrows; parental 293Luc cells are shown as a control. RLU, relative luminescence unit.
(TIF)

S4 Fig Sequences of NOD1 (A) and NOD2 (B) genomic target sites in 293Luc, 293Luc-ΔNOD1, 293Luc-ΔNOD2 and 293Luc-ΔNOD1/2 cells. Genomic target sites were PCR-amplified and cloned into pJet1.2/Blunt, and 6–8 plasmid clones from each cell type were sequenced. Bold numbers above the reference sequences indicate base positions in the coding sequences (CDS). sgRNA binding sites are underlined (for simplicity, the binding sites are shown as if located on the same DNA strand). ^ are Cas9n cut sites predicted according to [27]. Insertions are indicated by arrows, deletions by (-). Note that the large 90-bp insertion in allele 2 of 293Luc-ΔNOD1 cells is a triplicate copy of a 30-bp sequence next to one putative Cas9n cut site, whereas the 7-bp insertion is a single copy of a 7-bp sequence next to the other Cas9n cut site (gray boxes). The *NOD1* gene in the 293Luc-ΔNOD1/2 cells was not sequenced, assuming *NOD1* mutations to be identical to those in the parental 293Luc-ΔNOD1 cells.
(TIF)

S5 Fig luc2P activity in 293Luc cells transfected with NOD1 or NOD2 expression plasmids together with the muropeptides at indicated concentrations (same data as in Fig 4, but calculated as percent response to TNF). Mean ± s.d.
(TIF)

S6 Fig Establishment of optimal conditions for siRNA-mediated knock-down of NOD1 and NOD2 in human monocyte-derived macrophages. A, representative histogram plots of macrophages seeded at 2x10^4^ cells/well in 96-well plates and treated for 15 hrs with Lipofectamine 2000 alone (0.4 μl/well; left plot), fluorescein (FAM)-labeled scrambled siRNA alone (4 pmol/well; middle plot), or with equivalent amounts of complexes of Lipofectamine 2000 and FAM-siRNA prepared according to manufacturer’s instruction (right plot). FL-1 fluorescence was analysed using BD FACSCalibur flow cytometer (BD Biosciences, San Jose, CA). Percentages of fluorescein-positive cells are indicated. The marker was set according to untreated cells (<0.1% positive). B, macrophages were seeded in 96-well plates at different densities and treated for 15 hrs with different amounts of complexes of Lipofectamine 2000 + FAM-siRNA, whereafter analysed by flow cytometry as in A. Shown are mean fluorescence intensities (MFI) in the FL1-channel (one experiment out of 2 with similar results). Conditions resulting in highest siRNA uptake were selected for further experiments (arrow). C, macrophages were transfected with scrambled, NOD1 or NOD2 siRNAs (10^4^ cells, 8 pmol siRNA and 0.4 μl Lipofectamine 2000 per well) and harvested 48 hrs later. Relative expression of NOD1 and NOD2 mRNA was analysed by RT-PCR as described in Materials and Methods. NOD1 or NOD2 siRNAs caused around 50% reductions in the expression of target mRNAs. M ± s.d., n = 3; * p < 0.05 compared to expression of NOD1 or NOD2 in scrambled-transfected cells. D, macrophages were seeded at different densities, transfected with scrambled or NOD1 siRNAs (8 pmol siRNA and 0.4 μl Lipofectamine per well), and stimulated with a specific NOD1 agonist (iE-DAP; 50 μM) at indicated time points after transfection. TNF levels in the supernatants were measured by ELISA 12 hrs after addition of iE-DAP. Levels of TNF in NOD1-siRNA-transfected cultures were expressed as percentages of TNF levels in scrambled-siRNA-transfected cultures at the same time point and cell density. E, same experimental design as in D, except that cells were transfected with NOD2 siRNA and stimulated with MDP (1 μM). In D and E, shown is one experiment out of 2 with similar results. Based on these data, the 60-hr time point and cell density of 10^4^ per well was selected for further experiments.
(TIF)

S1 Table Single-stranded ODN sequences used in NOD1 and NOD2 KO experiments. (DOC)
