# Supplementary figures and images for: The Dual NOD1/NOD2 Agonism of Muropeptides Containing a Meso-Diaminopimelic Acid Residue
Source: PLoS One. 2016 Aug 11;11(8):e0160784. doi: 10.1371/journal.pone.0160784 (PMC4981496; doi:10.1371/journal.pone.0160784)

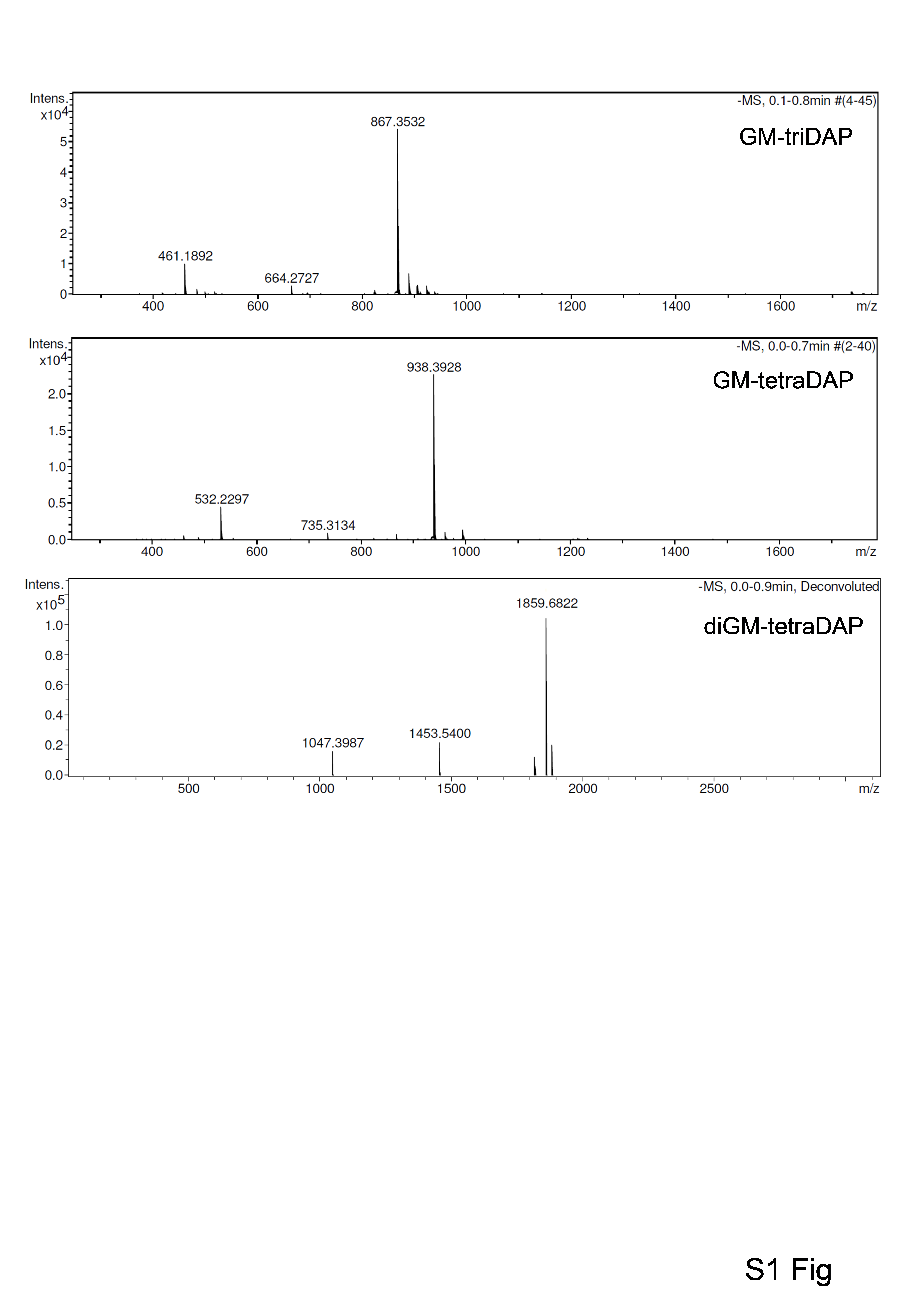

Supplement: S1 Fig — Each muropeptide is represented by a major peak corresponding to the native molecule and minor peaks corresponding to fragments devoid of one or more GlcNAc residues (these being GlcNAc residues per se or parts of MurNAc residues). Measured (calculated) molecular masses for [M–H]−ions are as follows: GM-triDAP native, 867.35 (867.56); GM-triDAP–GlcNAc, 664.27 (664.48); GM-triDAP– 2 GlcNAc, 461.19 (461.39); GM-tetraDAP native, 938.39 (938.64); GM-tetraDAP–GlcNAc, 735.31 (735.57); GM-tetraDAP– 2 GlcNAc 532.23 (532.5); diGM-tetraDAP native, 1859.68 (1859.25); diGM-tetraDAP– 2 GlcNAc, 1453.54 (1453.12), diGM-tetraDAP– 4 GlcNAc, 1047.4 (1047.97). Small peaks adjacent to some major peaks correspond to [M–2H+Na]−and [M–3H+2Na]−ions. In all three preps, no peaks corresponding to MDP ([M–H]–, 492.3) or glucosaminyl muramyl dipeptide (GMDP, [M–H]–, 695.5) were detected. (TIF) [file pone.0160784.s001.tif]

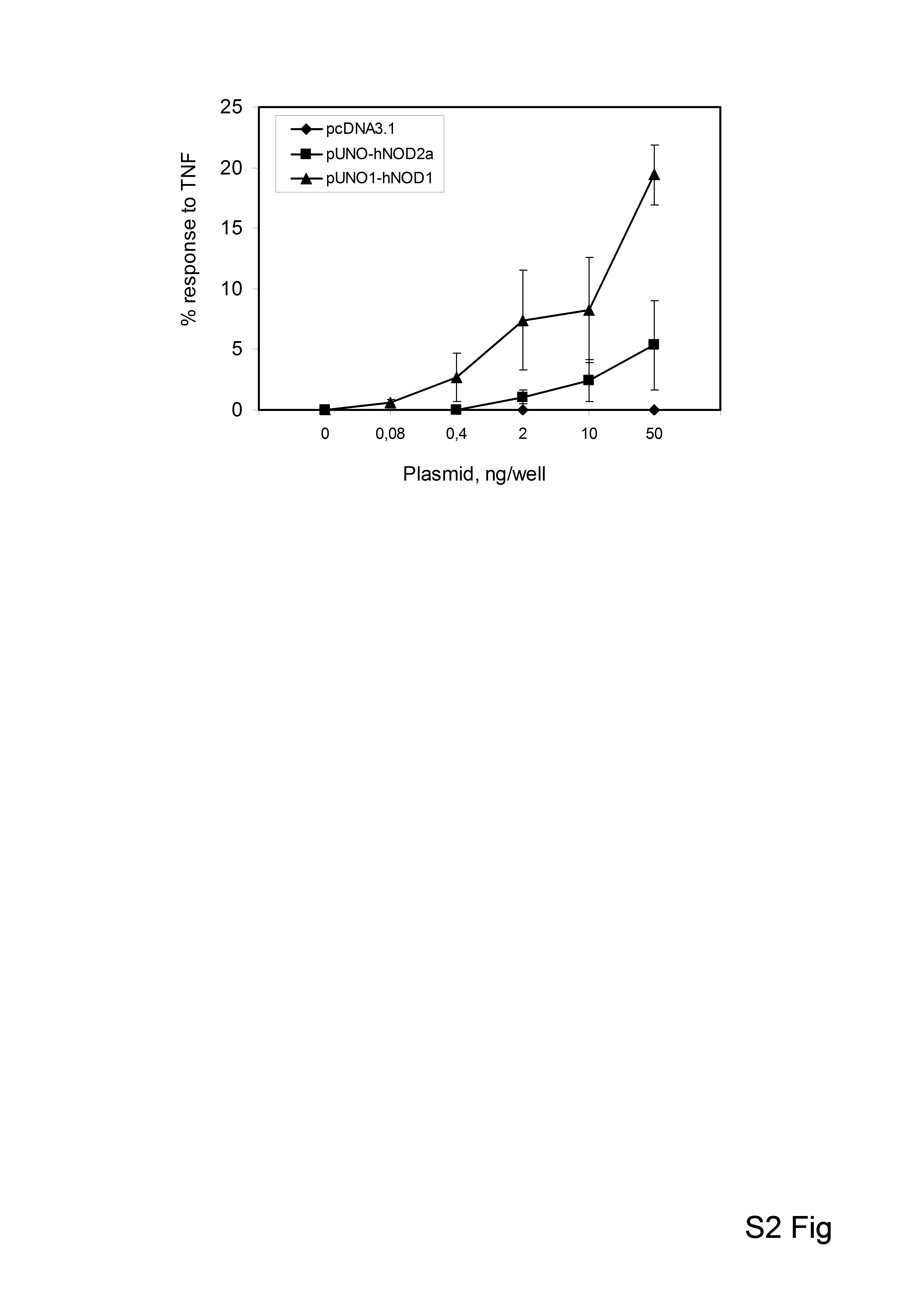

Supplement: S2 Fig — 293Luc cells were seeded in 96-well plates and transfected with the indicated doses of pUNO1-hNOD1, pUNO-hNOD2a or pcDNA3.1, and luc2P activity was measured 24 hrs later. Mean ± s.d., n = 3. (TIF) [file pone.0160784.s002.tif]

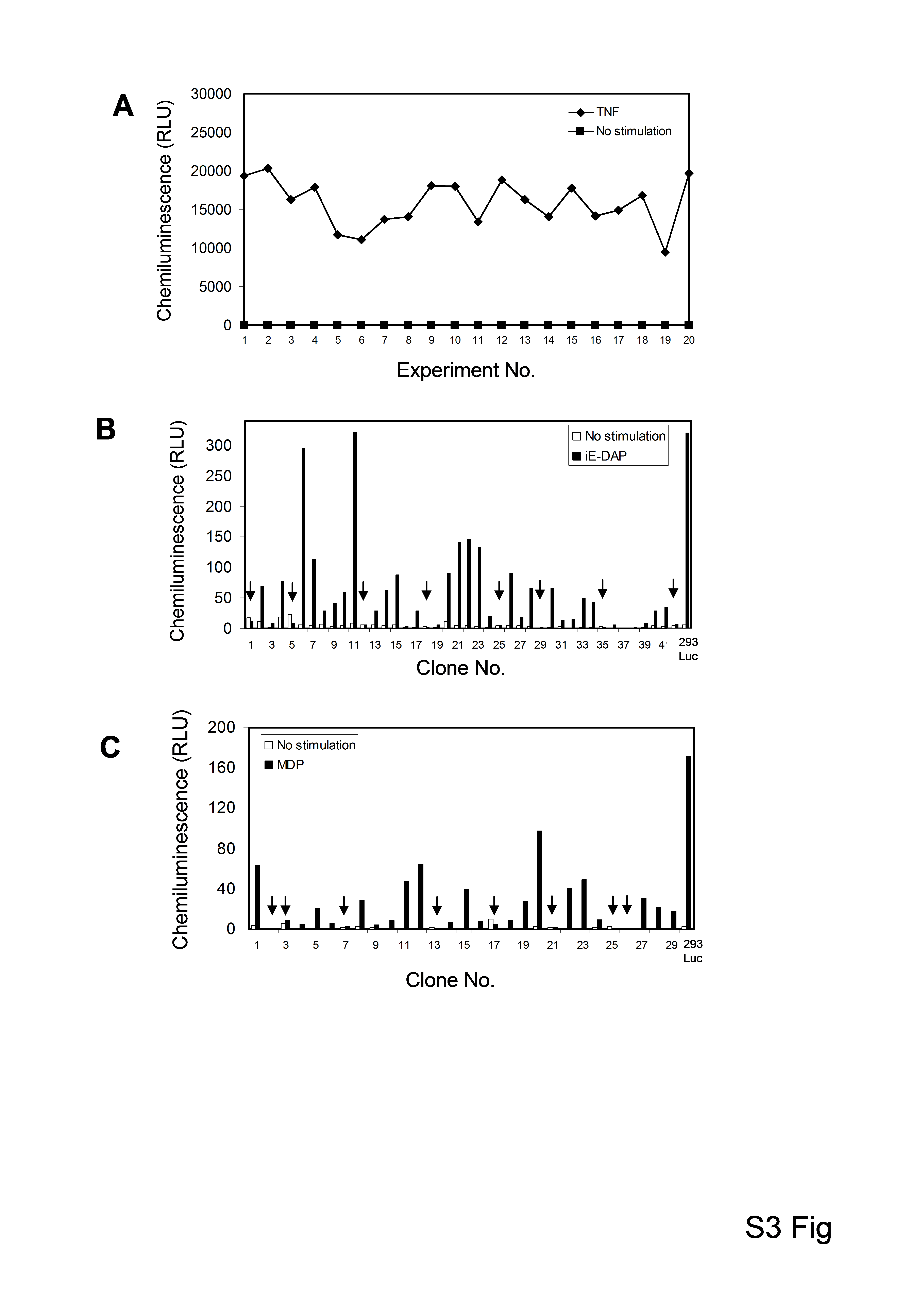

Supplement: S3 Fig — A, luc2P activity in 293Luc cells stimulated by TNF (100 ng/ml) for 24 hrs in 20 independent experiments performed over a two-year period. Shown are means of duplicates. B and C, 293Luc-derived clones with the putative NOD1 (B) or NOD2 (C) gene knock-out were stimulated by iE-DAP at 300 μM (B) or MDP at 1 μM, and luc2P activity was measured in 24 hrs. Non-responding clones (stimulation index less than 2) are marked by arrows; parental 293Luc cells are shown as a control. RLU, relative luminescence unit. (TIF) [file pone.0160784.s003.tif]

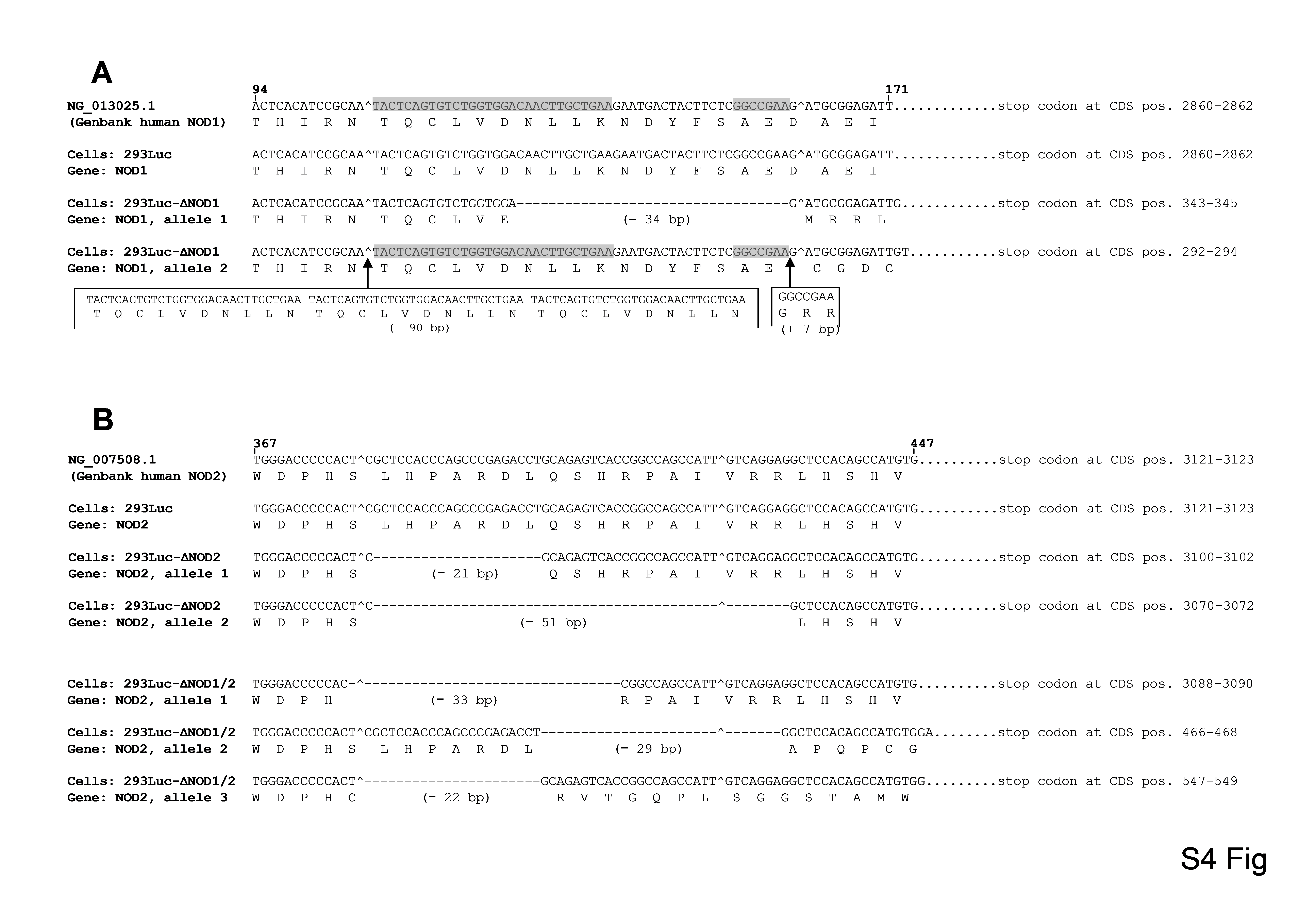

Supplement: S4 Fig — Sequences of NOD1 (A) and NOD2 (B) genomic target sites in 293Luc, 293Luc-ΔNOD1, 293Luc-ΔNOD2 and 293Luc-ΔNOD1/2 cells. Genomic target sites were PCR-amplified and cloned into pJet1.2/Blunt, and 6–8 plasmid clones from each cell type were sequenced. Bold numbers above the reference sequences indicate base positions in the coding sequences (CDS). sgRNA binding sites are underlined (for simplicity, the binding sites are shown as if located on the same DNA strand). ^ are Cas9n cut sites predicted according to [27]. Insertions are indicated by arrows, deletions by (-). Note that the large 90-bp insertion in allele 2 of 293Luc-ΔNOD1 cells is a triplicate copy of a 30-bp sequence next to one putative Cas9n cut site, whereas the 7-bp insertion is a single copy of a 7-bp sequence next to the other Cas9n cut site (gray boxes). The NOD1 gene in the 293Luc-ΔNOD1/2 cells was not sequenced, assuming NOD1 mutations to be identical to those in the parental 293Luc-ΔNOD1 cells. (TIF) [file pone.0160784.s004.tif]

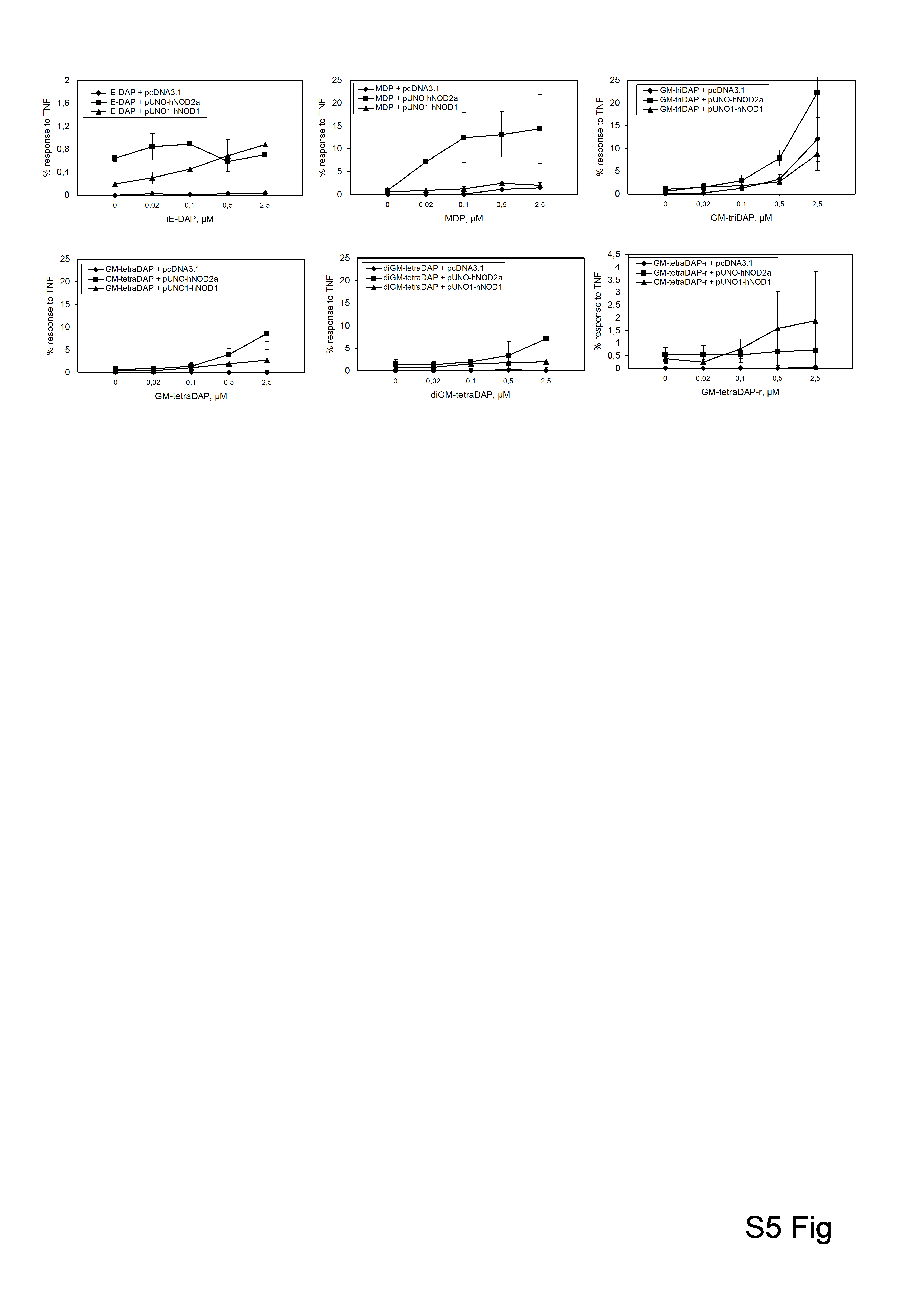

Supplement: S5 Fig — Mean ± s.d. (TIF) [file pone.0160784.s005.tif]

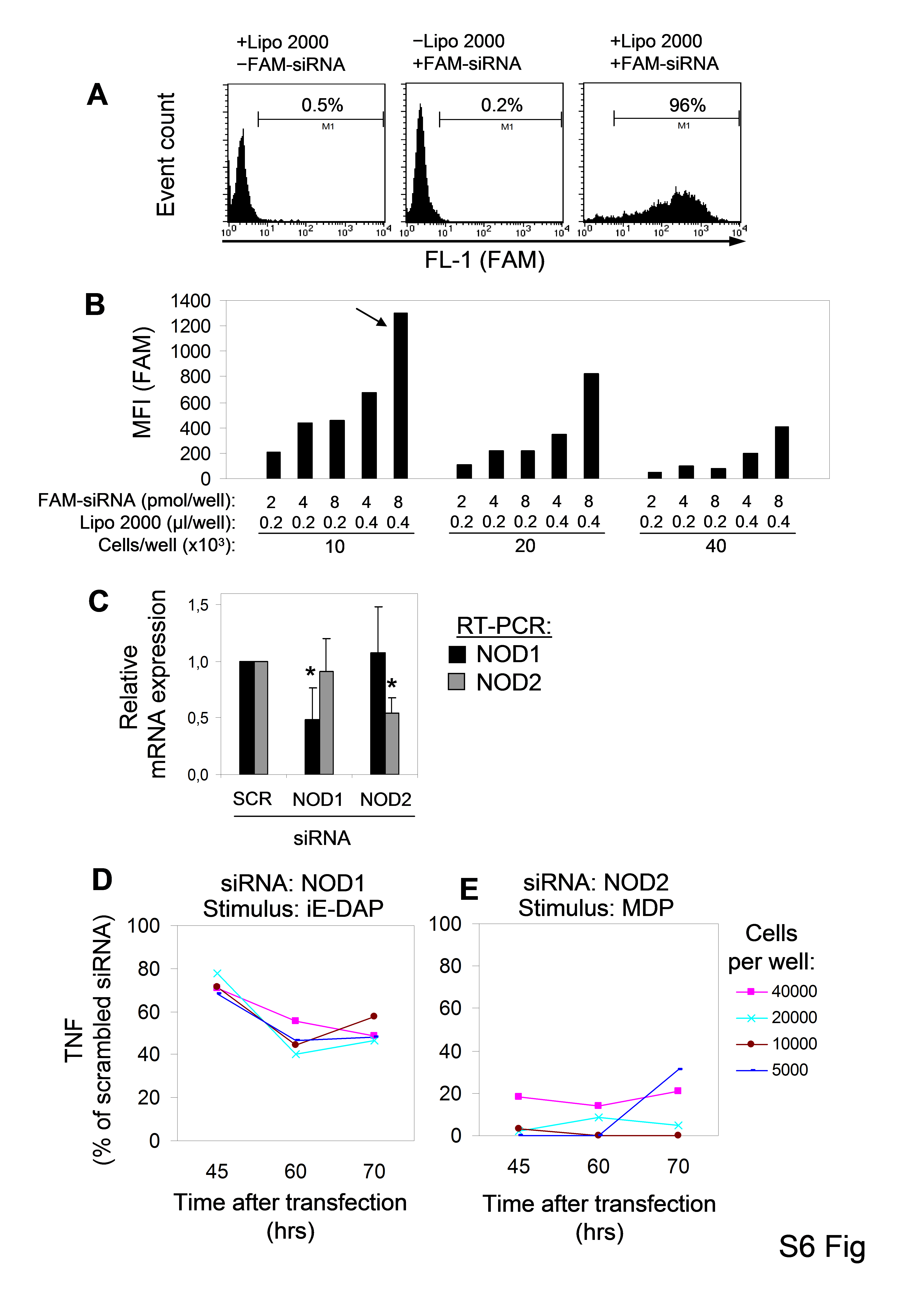

Supplement: S6 Fig — A, representative histogram plots of macrophages seeded at 2x10^4^ cells/well in 96-well plates and treated for 15 hrs with Lipofectamine 2000 alone (0.4 μl/well; left plot), fluorescein (FAM)-labeled scrambled siRNA alone (4 pmol/well; middle plot), or with equivalent amounts of complexes of Lipofectamine 2000 and FAM-siRNA prepared according to manufacturer’s instruction (right plot). FL-1 fluorescence was analysed using BD FACSCalibur flow cytometer (BD Biosciences, San Jose, CA). Percentages of fluorescein-positive cells are indicated. The marker was set according to untreated cells (<0.1% positive). B, macrophages were seeded in 96-well plates at different densities and treated for 15 hrs with different amounts of complexes of Lipofectamine 2000 + FAM-siRNA, whereafter analysed by flow cytometry as in A. Shown are mean fluorescence intensities (MFI) in the FL1-channel (one experiment out of 2 with similar results). Conditions resulting in highest siRNA uptake were selected for further experiments (arrow). C, macrophages were transfected with scrambled, NOD1 or NOD2 siRNAs (104 cells, 8 pmol siRNA and 0.4 μl Lipofectamine 2000 per well) and harvested 48 hrs later. Relative expression of NOD1 and NOD2 mRNA was analysed by RT-PCR as described in Materials and Methods. NOD1 or NOD2 siRNAs caused around 50% reductions in the expression of target mRNAs. M ± s.d., n = 3; * p < 0.05 compared to expression of NOD1 or NOD2 in scrambled-transfected cells. D, macrophages were seeded at different densities, transfected with scrambled or NOD1 siRNAs (8 pmol siRNA and 0.4 μl Lipofectamine per well), and stimulated with a specific NOD1 agonist (iE-DAP; 50 μM) at indicated time points after transfection. TNF levels in the supernatants were measured by ELISA 12 hrs after addition of iE-DAP. Levels of TNF in NOD1-siRNA-transfected cultures were expressed as percentages of TNF levels in scrambled-siRNA-transfected cultures at the same time point and cell density. E, same experimenta [file pone.0160784.s006.tif]
