# Supplementary material for: The Dual NOD1/NOD2 Agonism of Muropeptides Containing a Meso-Diaminopimelic Acid Residue
Source: PLoS One. 2016 Aug 11;11(8):e0160784. doi: 10.1371/journal.pone.0160784 (PMC4981496; doi:10.1371/journal.pone.0160784)
Supplement: S1 Table — (DOC) [file pone.0160784.s007.doc]

**S1 Table.** Single-stranded ODN sequences used in NOD1 and NOD2 KO experiments.

| Target  gene | Sequence ID  (genomic /  mRNA) | Sense* | Antisense* | Target site  (bases)** |
| --- | --- | --- | --- | --- |
| NOD1 | NG_013025.1 /  NM_006092.2 | CACCGCCACCAGACACTGAGTATTG | AAACCAATACTCAGTGTCTGGTGGC | 124-105 |
|  |  | CACCGCTACTTCTCGGCCGAAGATG | AAACCATCTTCGGCCGAGAAGTAGC | 144-163 |
| NOD2 | NG_007508.1 /  NM_022162.2 | CACCGTCGGGCTGGGTGGAGCGAGT | AAACACTCGCTCCACCCAGCCCGAC | 396-377 |
|  |  | CACCGTCACCGGCCAGCCATTGTC | AAACGACAATGGCTGGCCGGTGAC | 407-426 |

* “Sense” and “Antisense” refer to the plasmid strands, not to genomic DNA strands. Single-underlined letters are 5’-overhangs for sticky-end ligation into pKS-gRNA-BB vector. Double-underlined are guanines (and cytosines on the opposite strand) not in the original sequence, added to enhance the efficiency of sgRNA transcription (25).

** Base numbers refer to the gene coding sequences.
